# Infrared and Raman spectroscopic features of plant cuticles: a review

**DOI:** 10.3389/fpls.2014.00305

**Published:** 2014-06-25

**Authors:** José A. Heredia-Guerrero, José J. Benítez, Eva Domínguez, Ilker S. Bayer, Roberto Cingolani, Athanassia Athanassiou, Antonio Heredia

**Affiliations:** ^1^Nanophysics, Istituto Italiano di TecnologiaGenova, Italy; ^2^Instituto de Ciencias de Materiales de Sevilla, CSIC-USSeville, Spain; ^3^Instituto de Hortofruticultura Subtropical y Mediterránea La Mayora, CSIC-UMAMálaga, Spain; ^4^Istituto Italiano di TecnologiaGenova, Italy; ^5^Departamento de Bioquímica y Biología Molecular, Facultad de Ciencias, Universidad de MálagaMálaga, Spain

**Keywords:** plant cuticle, cuticle components, cuticle structure, infrared spectroscopy, Raman spectroscopy

## Abstract

The cuticle is one of the most important plant barriers. It is an external and continuous lipid membrane that covers the surface of epidermal cells and whose main function is to prevent the massive loss of water. The spectroscopic characterization of the plant cuticle and its components (cutin, cutan, waxes, polysaccharides and phenolics) by infrared and Raman spectroscopies has provided significant advances in the knowledge of the functional groups present in the cuticular matrix and on their structural role, interaction and macromolecular arrangement. Additionally, these spectroscopies have been used in the study of cuticle interaction with exogenous molecules, degradation, distribution of components within the cuticle matrix, changes during growth and development and characterization of fossil plants.

*If I am to know an object, though I need not know its external properties, I must know all its internal properties*.

Ludwig Wittgenstein (Tractatus Logico-Philosophicus, 1922)

## Introduction

**Figure 1:**
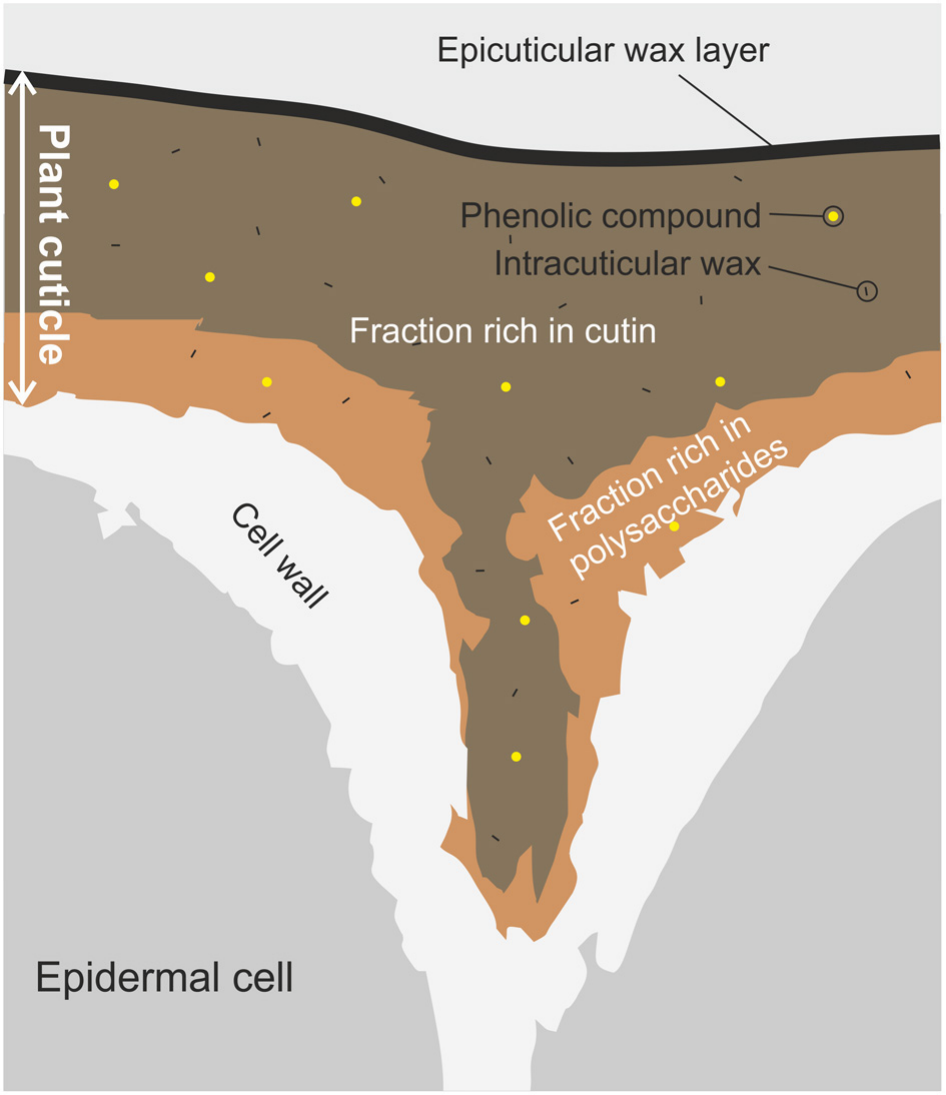
**Schematic diagram of a transverse section of a plant cuticle**. The figure shows the typical shape of the plant cuticle between two epidermal cells. Main components and their space distribution are displayed: the layer below is rich in polysaccharides from the cell wall, while the top layer is mainly constituted by cutin with a last layer of epicuticular waxes. Intracuticular waxes and phenolic compounds are spread through the plant cuticle.

The plant cuticle is the most external and continuous membrane that covers epidermal cells of leaves, fruits, petals, and non-lignified stems (Heredia, 2003). It is a composite membrane with a heterogeneous spatial distribution, Figure 1. The matrix is composed of cutin, a long-chain and insoluble polymer formed by hydroxylated and epoxy-hydroxylated C_16_ and C_18_ esterified fatty acids. The inner side is rich in polysaccharides (cellulose, hemicelluloses, and pectins) from the plant cell wall, and represents the attachment site to the outer epidermal cell wall. Other cuticle components are soluble waxes (mixtures of homologous series of long-chain aliphatics, such as alkanes, alcohols, aldehydes, fatty acids and esters, together with variable amounts of cyclic compounds such as triterpenoids) located on the surface (epicuticular waxes) or distributed through the cuticle (intracuticular waxes), and phenolic compounds such as cinnamic acids and flavonoids. Cuticles from some species may contain an alternative, and also chemically inert, polymer known as cutan, which is thought to consist of an ether-linked network of methylene chains, double bonds, and carboxyl groups (Villena et al., 1999; Jeffree, 2006). Cutan can partially or completely substitute cutin as the cuticle matrix. Significant differences in cuticle composition can be observed among plants, different organs within a plant or even among developmental stages of a given organ. Similarly, environmental conditions can modify the amount and composition of the cuticle (Domínguez et al., 2012). More details about the chemical composition and the spatial distribution of cuticle components can be found elsewhere (Jeffree, 2006; Pollard et al., 2008).

The cuticle is one of the most important plant barriers. In this sense, the biophysical properties of plant cuticles, structural, thermal, biomechanical, and hydric, are a complex balance between their protective role and the necessity of the plant to grow and develop (Domínguez et al., 2011). The main function ascribed to the cuticle is the protection of plants against uncontrolled water loss (Burghardt and Riederer, 2006). Additionally, as an interface between the plant and the environment, it has other secondary roles (Yeats and Rose, 2013): it represents the first defense against pests and pathogens, it can efficiently reflect dangerous UV light (depending on the crystallinity of the epicuticular waxes), and it is involved in the establishment of organ boundaries during development. Plants with superhydrophobic cuticles (Lotus effect) have further biological advantages in terms of self-cleaning and reduction of water content in the surface. The self-cleaning of cuticles can provide an additional defense against the deposition of pathogens and sunlight-blocking particles, while the reduction of water can slow down the growth of microorganism and the leaching of nutrients (Koch and Barthlott, 2009; Yeats and Rose, 2013).

In addition to the biological and agricultural importance of this plant barrier, its applied use as a source of organic compounds and its importance as plant biomass have recently begun to be considered. In this regard, cuticle components are a potential alternative feedstock for aliphatic compounds commonly found in oil plants (Tsubaki and Azuma, 2013). Thus, the potential applied value of tomato fruit peel, grape skins and green tea residues have been assessed (Arrieta-Baez et al., 2011; Mendes et al., 2013; Tsubaki and Azuma, 2013). Some of these aliphatic compounds, specifically cutin monomers, have been used to synthetize long-chain polyesters, resulting in polymers with similar characteristics to the plant cutin (Benítez et al., 2004a; Heredia-Guerrero et al., 2009; Gómez-Patiño et al., 2013; Vilela et al., 2014).

The lipid composition of plant cuticles is commonly determined by gas chromatography in combination with mass spectroscopy or flame ionization. Usually, to improve the resolution, hydroxyl and carboxylic acids functional groups are derivatized into the corresponding trimethyl silyl ethers and esters, using silylation reagents like bis-*N,O*-trimethylsilyltrifluoroacetamide (Walton and Kolattukudy, 1972; Jetter et al., 2006). In general, the chemical information provided by these techniques is considered very accurate both in the identification and in the quantification of these substances. However, these techniques present limitations: the identification of the components is not always complete, it is not possible to distinguish some functional groups after depolymerization (e.g., ester/carboxylic acid/carboxylate functional groups), and non-degradable fractions cannot be analyzed (Pollard et al., 2008). Additionally, they have substantial weaknesses regarding the structural determination of such components. More traditional structural techniques as X-ray diffraction have scarcely been used due to the amorphous nature of the plant cuticle (Luque et al., 1995). In contrast, solid state ^13^C nuclear magnetic resonance, using cross or direct polarization and magic-angle spinning methods, allows the identification of functional groups and structures, the quantification of the molecular dynamics and the assessment of the cross-linking capability. Nonetheless, these spectroscopic measurements provide limited information concerning molecular structure due to overlapping of the broad spectral lines and, for quantitative measurements, long acquisition times are required (Serra et al., 2012). IR and Raman spectroscopies are non-destructive and accessible techniques which have shown important advantages in the chemical and structural analysis of plant cuticles, e.g., identification of functional groups and conformations, determination of intra- and intermolecular interactions of cuticle components with exogenous molecules, and qualitative measurements of the cutin polymerization. These spectroscopies are based on the excitation of the molecular vibrations of chemical bonds by the absorption of light (infrared spectroscopy) or the inelastic scattering of photons (Raman spectroscopy). Both phenomena are governed by different mechanisms, affecting the exact position, the appearance and intensity of the bands in the corresponding spectra. Main advantages of Raman spectroscopy are the possibility of using water as solvent and practically no sample preparation. Nevertheless, this spectroscopy presents some drawbacks. For example, fluorescence may interfere with the mechanism of the Raman effect and overlap the signals. On the other hand, IR measurements are fast, easy and no interferences are produced by other mechanisms. However, IR spectroscopy is very sensitive to water and it cannot be used as solvent. Also, the preparation of samples for IR spectroscopy presents some limitations on sample thickness, uniformity and dilution to avoid saturation. Furthermore, the wide set of different modes of acquisition of infrared and Raman techniques can provide important and complementary chemical information. For instance, in the transmission mode the sample can be placed directly into the path of the infrared beam, providing information about whole system. Other mode of acquisition is ATR-FTIR. ATR is a useful technique to obtain the IR spectrum of the surface of samples. The sample is placed in contact with an internal reflection element, a material with a high refractive index, the light is totally reflected several times and the surface of the sample interacts with the evanescent wave resulting in the absorption of radiation at each point of reflection. Other interesting feature of IR and Raman spectroscopies is the possibility of coupling to microscopes (microspectroscopy), allowing the study of structures in specific regions of a histological section. More information about the different IR and Raman techniques can be found elsewhere (Laserna, 1996; Günzler and Gremlich, 2002).

In this review, we summarize the main applications of infrared and Raman spectroscopies in the characterization of the plant cuticles. First, the spectral characterization of plant cuticles and their components is described in terms of assignments, relationships between bands, interactions and structure. In the second part, main applications of these spectroscopies, such as characterization of the plant cuticle during development and degradation, interaction with exogenous molecules, characterization of fossilized plant cuticles, and the chemical imaging of specific components, are reviewed.

## Characterization of plant cuticles and cuticle components

### Plant cuticles

The characterization of plant cuticles by IR and Raman spectroscopies have provided significant information on the nature of functional groups present in the cuticle matrix and on the structural role, interaction and macromolecular arrangement of their components. For an introduction to the topic see Chamel and Maréchal (1992), Ramírez et al. (1992), Villena et al. (2000), Ribeiro da Luz (2006).

**Figure 2:**
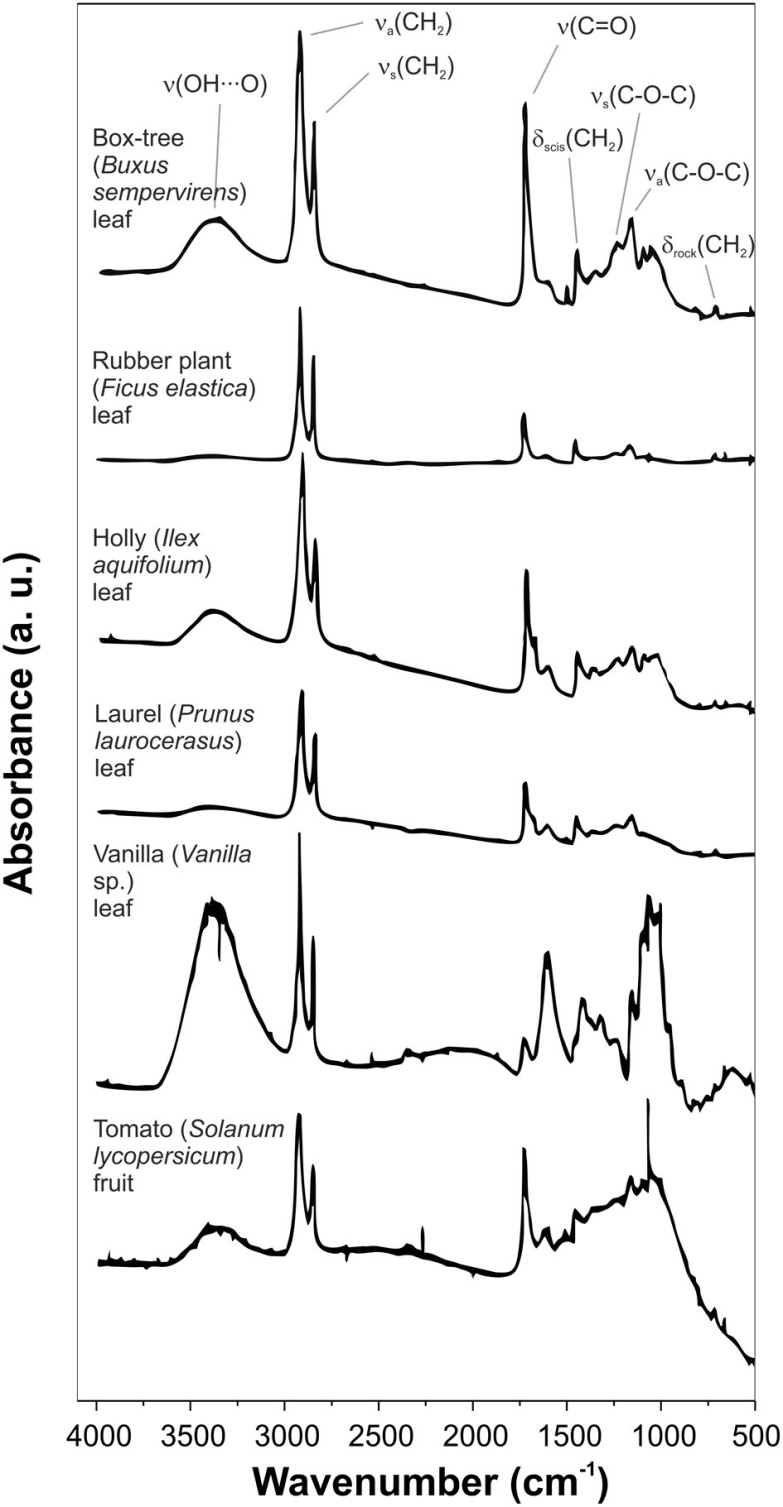
**Transmission FTIR spectra of isolated cuticles of different plant species**. Some main bands are assigned. Adapted from Chamel and Maréchal (1992).

FTIR spectra analysis of isolated cuticles from different species allowed the identification of several bands characteristic of plant cuticles, Figure 2 (Chamel and Maréchal, 1992; Ramírez et al., 1992; España et al., 2014):

– A broad band around 3400 cm^−1^ assigned to the stretching vibration of hydroxyl groups that interact by H bonding, ν (O-H···O). The intensity of this band depended on the plant species. The polysaccharide fraction and, secondly, the non-esterified hydroxyl groups of cutin were considered the major contributors to this band.
– Two strong bands at approximately 2920 and 2850 cm^−1^ assigned to the asymmetrical and symmetrical stretching vibrations of CH_2_ groups, ν_*a*_(CH_2_) and ν_*s*_(CH_2_) respectively, accompanied by the corresponding δ (CH_2_) bending vibrations at around 1468, 1313, and 725 cm^−1^. These bands were ascribed to the aliphatic material present in the plant cuticle: cutin, waxes and cutan.
– A strong band at about 1730 cm^−1^ corresponding to ν (C=O) stretching ester vibration accompanied by two bands at around 1167 and 1104 cm^−1^ attributed to asymmetrical and symmetrical C-O-C stretching ester vibrations. These bands were associated with the cutin matrix.

In addition to these main bands, other minor absorptions can be observed:

– Shoulders on the ν (C=O) band, usually about 1715, 1705, and 1685 cm^−1^. These vibrations were associated with ester and carboxylic acid groups with different interactions by H bonding (for more details see section Cutin).
– Bands in the 1650-1500 cm^−1^ spectral region with variable intensity depending on the plant species. They were related to aromatic and C=C functional groups from phenolic compounds or cutan.
– A weak band at 1271 cm^−1^ assigned to δ (OH) bending vibrations of hydroxyl groups from polysaccharides and cutin.

These assignations have been also used in the study of non-isolated plant cuticles of seeds (Sugiura et al., 2009; Yan et al., 2009), stems (Himmelsbach and Akin, 1998; Himmelsbach et al., 1999), fibers (Morrison III et al., 1999) and epidermal cells (Stewart, 1996).

In the Table 1 a detailed band assignment of transmission and ATR-FTIR spectra of isolated tomato fruit cuticles (*Solanum lycopersicum* L.) is shown (Ramírez et al., 1992; España et al., 2014). In general, most intense bands corresponded to the aliphatic and ester groups of cutin, the main component of these cuticles. Main differences between red ripe and immature green stages of growth were ascribed to the aromatic rings and double bonds of phenolics. The spatial and asymmetrical distribution of cuticle components was characterized by ATR-FTIR. Bands associated with waxes and cutin were stronger in the spectrum of the cuticle outer surface, while in the spectrum of the inner surface the absorptions assigned to polysaccharides were more intense.

**Table 1:** **Main functional groups assigned to the different vibrations present in the transmission (red ripe stage) and ATR-FTIR (immature green and red ripe stages) spectra of tomato (*Solanum lycopersicum*) fruit cuticle (adapted from Ramírez et al., 1992; España et al., 2014)**.

**Assignment^a^**	**Wavenumber (cm^-1^) (Intensity^b^)**					**Cuticle component^d^**
	**Transmission FTIR**	**ATR-FTIR**				
	**Red ripe^c^**	**Immature green^c^**		**Red ripe^c^**		
	**Cuticle**	**Cuticle outer face**	**Cuticle inner face**	**Cuticle outer face**	**Cuticle inner face**	
ν (O-H···O)	3347 (m, b)	3390 (w, b)	3340 (s, b)	3304 (m, b)	3343 (s, b)	Cutin, polysaccharides
ν_a_(CH_2_)	2927 (vs)	2919 (vs)	2921 (s)	2918 (vs)	2922 (s)	Cutin, waxes
ν_s_(CH_2_)	2852 (s)	2850 (s)	2852 (s)	2849 (s)	2853 (s)	Cutin, waxes
ν (C=O) ester	1731 (s)	1730 (s)	1728 (s)	1731 (s)	1728 (s)	Cutin
ν (C=O···H) ester	1713 (m, sh)	–	–	–	–	Cutin
ν (C=O···H weak) acid	–	1707 (m, sh)	1706 (m, sh)	1707 (m, sh)	1700 (m, sh)	Cutin
ν (C=O···H strong) acid	–	1687 (w, sh)	1685 (w)	1686 (w, sh)	1685 (w)	Cutin
ν (C=C) phenolic acid	1624 (m)	1635 (w)	1629 (w)	1628 (m)	1627 (s)	Phenolic compounds
ν (C-C) aromatic	1606 (s)	1605 (w)	1606 (w)	1605 (m)	1606 (m, sh)	Phenolic compounds
ν (C-C) aromatic (conjugated with C=C)	1551 (w)	1550 (vw)	1556 (vw)	1552 (w, b)	1555 (w, b)	Phenolic compounds
ν (C-C) aromatic (conjugated with C=C)	1515 (m)	1515 (w)	1514 (w)	1515 (m)	1515 (m)	Phenolic compounds
δ (CH_2_) scissoring	1463 (w)	1463 (m)	1457 (m)	1463 (m)	1457 (m)	Cutin, waxes
ν (C-C) aromatic (conjugated with C=C)	1440 (w)	1440 (w, sh)	1436 (m)	1438 (w, sh)	1437 (m)	Phenolic compounds
δ (CH_2_) wagging and twisting	1344 (w, b)	1367 (m, b)	1367 (m)	1360 (m, b)	1365 (m, b)	Cutin, waxes
δ (OH)	1278 (w)	1244 (m, b)	1243 (m, b)	1246 (m, b)	1243 (m, b)	Cutin, polysaccharides
ν_a_(C-O-C), ester	1167 (m)	1166 (s)	1161 (s)	1166 (vs)	1162 (s)	Cutin
ν_s_(C-O-C), ester	1103 (w)	1104 (m)	1101 (s)	1104 (m)	1101 (s)	Cutin
ν (C-O-C), glycosydic bond	–	1054 (w, b)	1053 (vs)	1060 (w)	1050 (vs)	Polysaccharides
ν (C-O)	984 (w, b)	967 (m, sh)	–	984 (w)	–	Cutin, polysaccharides
γ(C-H) aromatic	833 (w)	834 (w)	833 (w)	834 (m)	833 (w)	Phenolic compounds
δ (CH_2_) rocking	723 (w)	724 (m)	721 (m)	722 (m)	720 (w)	Cutin, waxes

a *ν, stretching; δ, bending; γ, out-of-plane bending; a, asymmetric; s, symmetric*.

b *s, strong; m, medium; w, weak; vs, very strong; vw, very weak; b, broad; sh, shoulder*.

c *Tomato fruits used in these measurements belonged to different cultivars and some different spectral features can be observed for this reason*.

d *Main contributions*.

**Figure 3:**
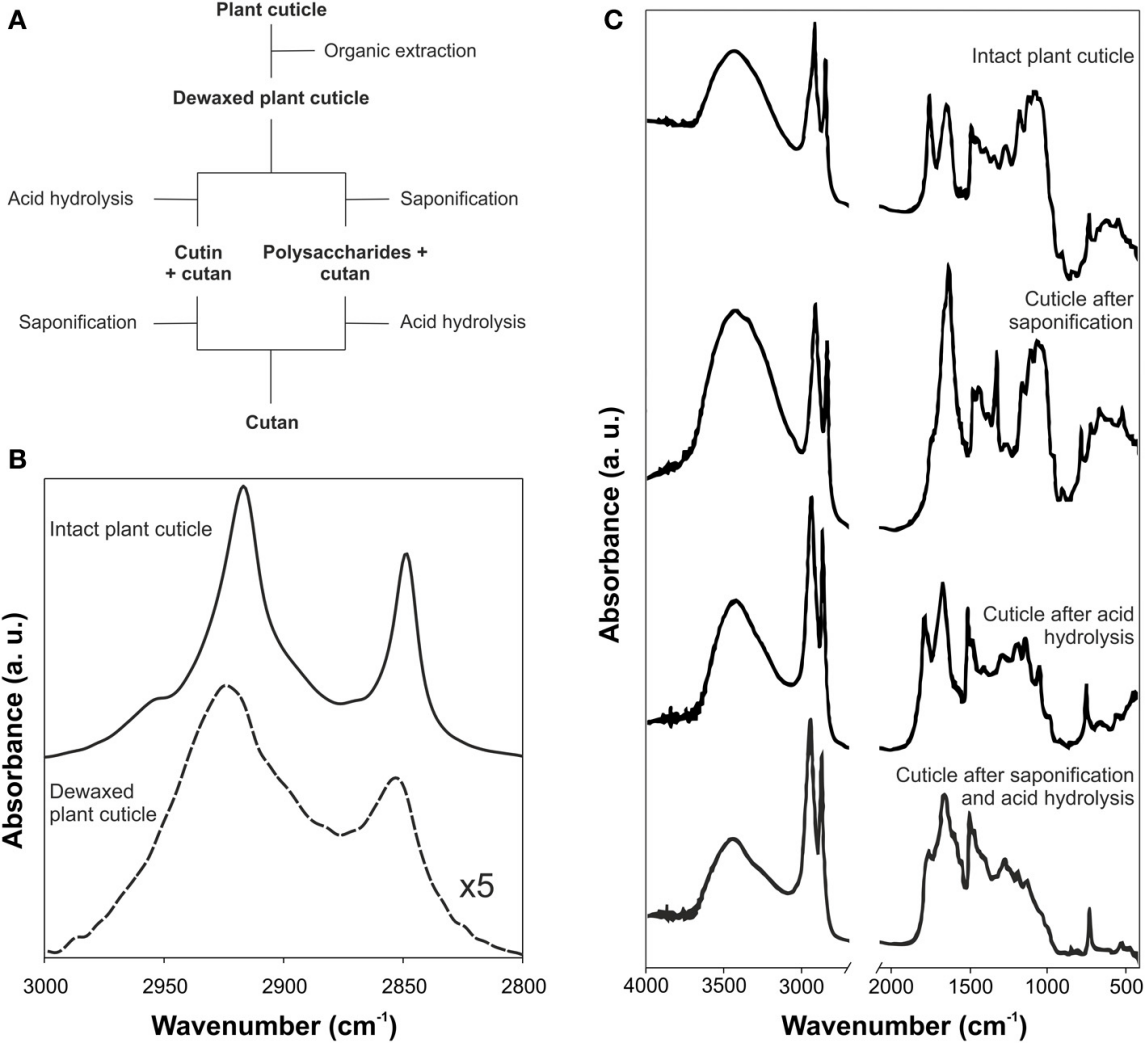
**(A)** Scheme for the sequential removing of cuticular components. **(B)** ATR-FTIR spectra of intact and dewaxed plant cuticle of peach fruit (adapted from Fernández et al., 2011, www.plantphysiology.com, Copyright American Society of Plant Biologists). **(C)** Transmission FTIR spectra of the isolated plant cuticle of *Clivia miniata* leaf after acid, basic or both treatments (adapted from Villena et al., 2000, with permission of Elsevier).

Besides the characterization of the isolated cuticle, IR was used to monitor the selective removal of each cuticle component (Villena et al., 2000; Johnson et al., 2007; Chen et al., 2008; Li et al., 2010; Fernández et al., 2011). The chemical removal of each fraction was accompanied by spectroscopic changes. Alternatively, polysaccharides and cutin matrixes were directly studied after extraction and depolymerization of the other cuticle components. A general scheme of the chemical procedures usually employed is shown in Figure 3A. Thus, cuticular waxes have been commonly removed by organic solvent extraction and, then, cutin or polysaccharides have been depolymerized by basic or acid hydrolysis, respectively. After wax extraction a reduction of the intensity of the bands associated with the C-H groups, in accordance to the amount and composition of such waxes, was observed. Figure 3B shows these changes in peach (*Prunus persica* (L.) Stokes) fruit cuticles (Fernández et al., 2011). The C-H stretching region of the intact cuticles displayed two strong absorptions (2917 and 2849 cm^−1^, ν_*a*_(CH_2_) and ν_*s*_(CH_2_), respectively) and two small shoulders (2954 and 2870 cm^−1^, ν_*a*_(CH_3_) and ν_*s*_(CH_3_), respectively), while in the de-waxed cuticle only two broader and shifted bands at 2925 and 2853 cm^−1^ corresponding to the methylene groups of the cutin matrix were present. Depolymerization by acid or basic hydrolysis produced stronger changes. In the case of *Clivia miniata* Regel leaf cuticles, Figure 3C, the bands associated with ester groups from the cutin disappeared after basic hydrolysis (mainly the absorption at around 1730 cm^−1^ ascribed to the stretching of C=O in ester groups), while polysaccharide bands were removed with acid treatment (δ (OH) and ν (C-O) bands of secondary hydroxyl groups in the region of 1100-950 cm^−1^) (Villena et al., 2000). The final residue, cutan, only showed an aliphatic and aromatic composition.

### Cutin

**Figure 4:**
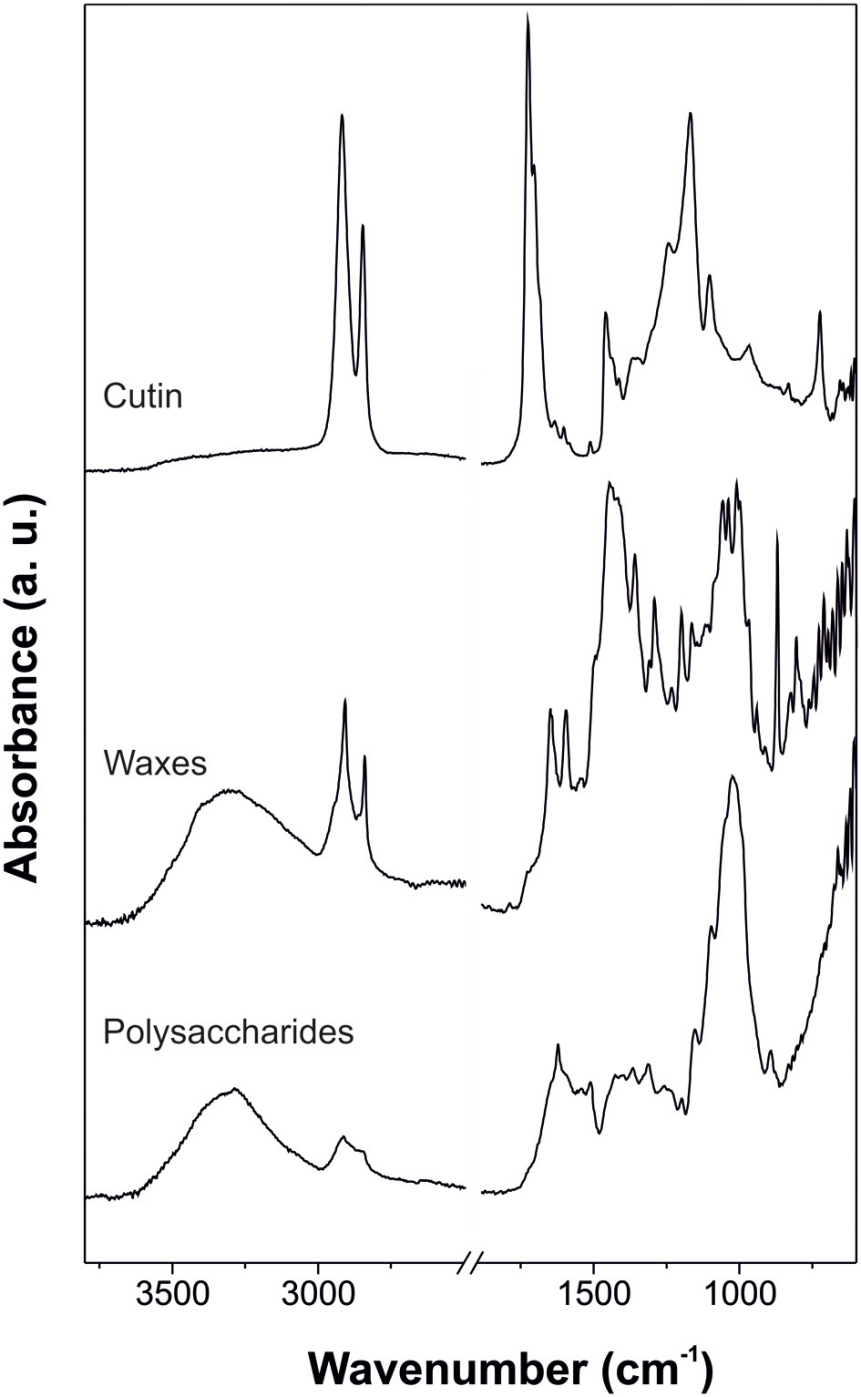
**ATR-FTIR spectra of cutin (adapted from España et al., 2014, with permission of John Wiley and Sons), waxes (adapted from Heredia-Guerrero et al., 2012, with permission of Elsevier), and polysaccharides (adapted from López-Casado et al., 2007) of isolated tomato fruit cuticle**.

The IR spectrum of cutin is characterized by its chemical structure: a polyester formed by polyhydroxy fatty acids. In the case of tomato fruit cutin, main bands were ascribed to hydroxyl (ν (O-H) at 3403 cm^−1^), methylene (mainly ν_*a*_(CH_2_) at 2926 cm^−1^, ν_*s*_(CH_2_) at 2854 cm^−1^, δ (CH_2_) scissoring at 1463 cm^−1^ and δ (CH_2_) rocking at 724 cm^−1^) and ester (ν (C=O) at 1729 cm^−1^, ν_*a*_(C-O-C) at 1169 cm^−1^ and ν_*s*_(C-O-C) at 1104 cm^−1^) functional groups (España et al., 2014). Figure 4 shows an ATR-FTIR spectrum of cutin where these bands can be easily identified.

The C=O ester stretching band is usually accompanied by different shoulders. The most common one is the absorption at around 1713 cm^−1^, indicative of interactions by H bonding of the ester group (Ramírez et al., 1992; Girard et al., 2012). Other authors have assigned this vibration to C=O groups of carboxylic acids which would appear as consequence of a putative hydrolysis of esters groups of the cutin after the acid treatment employed to remove polysaccharides, see Figure 3A (Maréchal and Chamel, 1996). However, this shoulder is also present in intact plant cuticles. Furthermore, shoulders at around 1705 and 1685 cm^−1^ have been detected (España et al., 2014) and assigned to carboxylic acid groups involved in weak H bonds and COOH interacting by strong H bonds, respectively.

Other important aspect in the characterization of cutin by IR spectroscopy is the ratio between the stretching bands of methylene and ester groups, this is, a relationship between the most repeated structural unit of the cutin (CH_2_ groups) and the bond of the different hydroxyl fatty acids (ester groups) that cross-link the cutin matrix. This ratio has been used for qualitative comparisons by several authors (even in isolated plant cuticles) and calculated in different forms (Benítez et al., 2004b; Chefetz, 2007; Girard et al., 2012; Heredia-Guerrero et al., 2012; España et al., 2014). We recommend the ratio of intensities ν (C=O)/ν_*a*_(CH_2_) and the denomination of “esterification index.” Thus, values of the “esterification index” are directly related to the cross-linking of cutin. High values of this ratio imply a higher esterification degree.

### Cutan

Cutan structure from *Agave americana* L. and *C. miniata* R. leaves have been studied by IR spectroscopy, Figure 3C “cuticle after saponification and acid hydrolysis” (Tegelaar et al., 1989; Villena et al., 1999). FTIR confirmed the polymethylenic/fatty acid nature of cutan with absorbances assigned to hydroxyl (ν (O-H) at around 3410 cm^−1^), methylene (ν_*a*_(CH_2_) at 2911 cm^−1^, ν_*s*_(CH_2_) at 2842 cm^−1^, δ (CH_2_) scissoring at 1474 cm^−1^ and δ (CH_2_) rocking at 731 cm^−1^), double bonds (ν (C=C) at 1650 cm^−1^), carboxylic acid (ν (O-H···HOOC) at 2693 cm^−1^ and ν (C=O) at 1730 cm^−1^) and carboxylate functional groups (ν_*a*_(COO^−^) at 1633 cm^−1^).

### Cuticular waxes

Cuticular waxes can be divided into crystalline and amorphous domains. Crystalline regions are arranged in an ordered structure of the aliphatic chains of the waxes, while amorphous zones are formed by chain ends, functional groups, short-chain aliphatics and non-aliphatic compounds (Riederer and Schreiber, 1995). These structural characteristics were observed by IR spectroscopy as well-defined bands from the aliphatic crystalline fraction (typical absorptions associated with methylene groups such as ν_*a*_(CH_2_), ν_*s*_(CH_2_), δ (CH_2_) scissoring and δ (CH_2_) rocking) with contributions of the functional groups that form the amorphous region: methyl (usually, ν_*a*_(CH_3_) and ν_*s*_(CH_3_) shoulders at higher wavenumbers of the corresponding bands for the CH_2_ groups), hydroxyl, ester, aldehyde, ketone, carboxylic acid, and aromatic groups (e.g., Dubis et al., 1999, 2001; Ribeiro da Luz, 2006; Johnson et al., 2007 and others). Figure 4 shows an ATR-FTIR spectrum of the reconstituted waxes of tomato fruit cuticle where the participation of crystalline and amorphous regions is observed.

ATR-FTIR spectroscopy has been also used to study the phase behavior and molecular structure of plant cuticular waxes of *Hedera helix* L., *Juglans regia* L. and *Malus x domestica* Borkh. using the above-mentioned bands assigned to methylene groups (Merk et al., 1998; Khanal et al., 2013). The position and shape of the ν_*a*_(CH_2_) and ν_*s*_(CH_2_) vibrations strongly depended on temperature: a shift to higher wavenumbers and an increase in band width was observed with higher temperatures. This behavior resulted from an increase in the number of gauche conformers, indicating a higher alkyl chain disorder with temperature. Peak doublets assigned to δ (CH_2_) scissoring and δ (CH_2_) rocking were ascribed to the orthorhombic crystal structure of aliphatic chains. These two peaks merged into a single band with temperature increase, typical of a transformation to a hexagonal structure and subsequent melting. Furthermore, the crystallinity of aliphatic chains was estimated by the ratio of peaks areas at 730 and 720 cm^−1^, respectively. On the other hand, the analysis of the phase behavior of pure 1-tetradecanol and 1-octanol and their binary mixtures by FTIR showed good spectroscopic similarities with the above described cuticular waxes (Carreto et al., 2002).

In addition, other molecules have been used as models of cuticular waxes. FTIR spectra of mixed monolayers of oleanolic acid, one of the most important triterpenoids present in the cuticle, and stearic acid have been carried out. Results showed that oleanolic acid (up to 0.4 mole fraction) did not perturb the all-*trans* conformation of the aliphatic chains of stearic acid (Teixeira et al., 2007). The ν (C=O) of oleanolic acid suggested that the carboxylic acid groups formed dimers. When oleanolic acid was combined with stearyl stearate, the resulting DRIFT spectrum was a superposition of the spectra of the single compounds, indicating the immiscibility of these substances (Teixeira et al., 2009).

Raman techniques have been also employed in the study of cuticular waxes. TIR-Raman spectroscopy, with a limited penetration depth (40 nm), was used to examine *in vivo* surface waxes of barley (*Hordeum vulgare* L.) leaves (Greene and Bain, 2005) while epicuticular waxes of mature mango (*Mangifera indica* L.) fruit were characterized by Raman spectroscopy (Prinsloo et al., 2004). A comprehensive analysis of the triterpenoid fraction of cuticular waxes was carried out by Raman microspectroscopy (Yu et al., 2007). This analysis resulted in the *in situ* detection of such molecules on *Prunus laurocerasus* L. leaf cuticles.

### Polysaccharides

Despite the importance of polysaccharides in the plant cuticle (López-Casado et al., 2007; Domínguez et al., 2011; Guzmán et al., 2014), a comprehensive assignation of the corresponding spectra of this cuticle fraction is missing. Usually, for the assignations of polysaccharide absorptions, comparisons with typical bands of pure cellulose or cell wall polysaccharides have been carried out. In general, cuticle polysaccharides are characterized by δ (OH) and ν (C-O) bands of secondary hydroxyl groups (Villena et al., 2000; Johnson et al., 2007). An ATR-FTIR spectrum of the polysaccharide fraction of tomato fruit cuticle is shown in the Figure 4.

### Phenolics

Phenolics have been characterized by IR spectroscopy in tomato fruit cuticles (Ramírez et al., 1992; España et al., 2014) and monitored during fruit development (see section Plant development for more details). In the 1650-1400 cm^−1^ spectral region, five bands were easily identified: ν (C=C) of phenolic acids (1624 cm^−1^), ν (C-C) aromatic (1606 cm^−1^), and three ν (C-C) aromatic conjugated with C=C (1551, 1515 and 1440 cm^−1^). Additionally, two absorptions at lower wavenumbers were detected: C-H and C-C out-of-plane bending vibrations at 833 and 518 cm^−1^, respectively. These two absorptions were associated with 1,4-disubstituted benzene molecules.

## Applications

### Plant development

The plant cuticle is a dynamic system whose chemical composition and, hence, its properties are changed during development. These chemical modifications are a source of spectral variability and have been characterized by IR and Raman spectroscopies.

**Figure 5:**
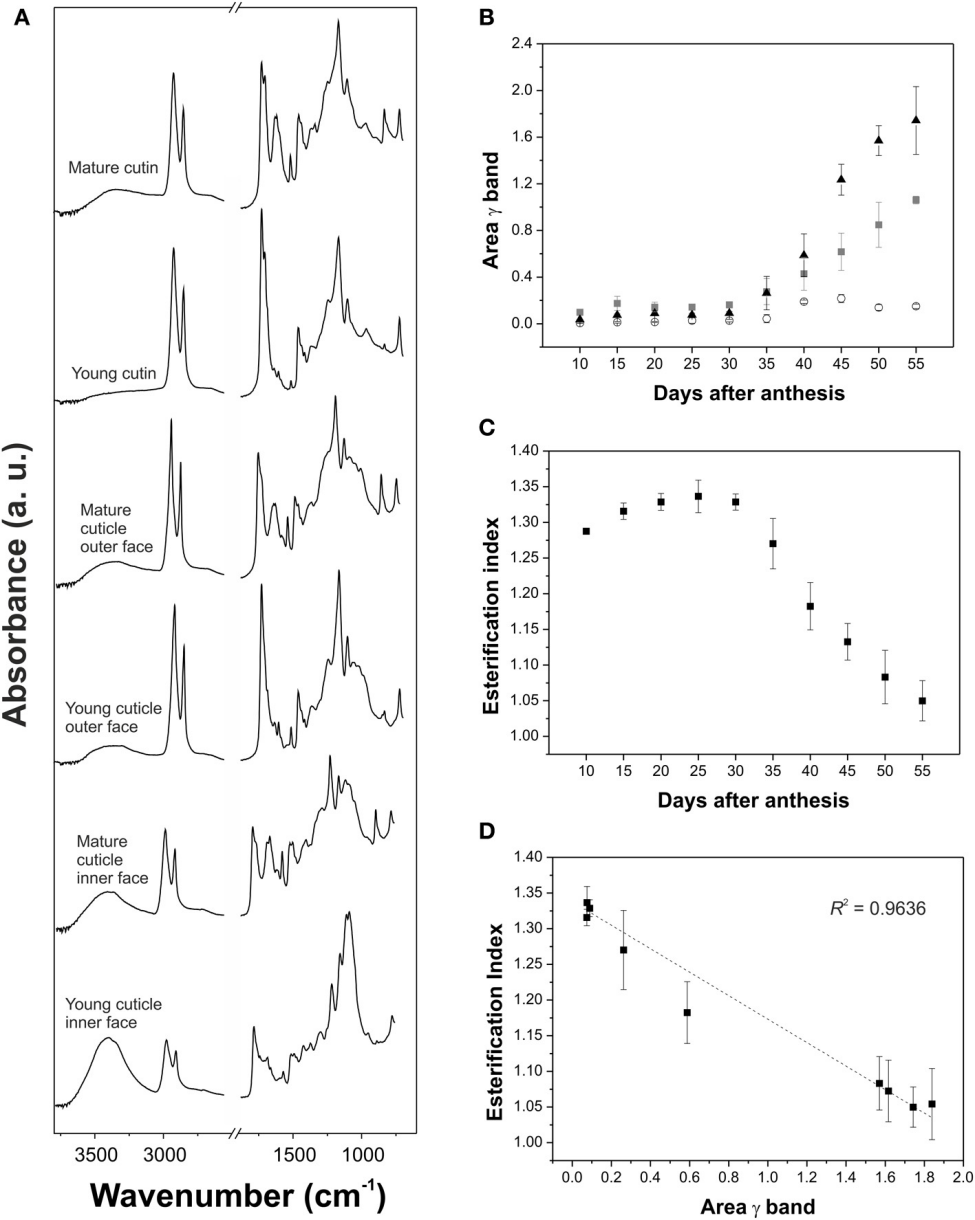
**(A)** ATR-FTIR spectra in the 3800-600 cm^−1^ region of young (15 daa) and mature (55 daa) cutin, cuticle outer face and cuticle inner face of tomato fruit. **(B)** Area of the γ (C-H) aromatic band associated with the presence of phenolic compounds in tomato fruit cuticle during development. Triangles: cutin; squares: cuticle outer surface; circles: cuticle inner surface. **(C)** Cutin esterification index, calculated as the ratio between the intensities of the C=O stretching vibration of the ester (1730 cm^−1^) and the asymmetric vibration of the methylene (2925 cm^−1^) functional groups, during fruit development in tomato fruit. **(D)** There was a linear relationship between the esterification index and γ band area in the cutin during fruit development. Adapted from España et al. (2014), with permission of John Wiley and Sons.

Analysis of the spectral changes during development has been carried out in isolated tomato fruit cuticles (Luque et al., 1995; Benítez et al., 2004b; España et al., 2014). Comparison of the FTIR spectra of immature green and red ripe tomato cuticles showed the appearance of absorptions at 1630, 1530, and 900-800 cm^−1^ in the cuticles of ripe tomatoes, which were ascribed to the functional groups of phenolic compounds and flavonoids (see section Phenolics for more information) (Luque et al., 1995; Benítez et al., 2004b). Additionally, changes in the esterification index of the isolated cutin were observed between these stages (Benítez et al., 2004b). A more thorough analysis of cuticle changes during tomato growth and ripening was performed by ATR-FTIR (España et al., 2014). Infrared spectra did not change significantly during growth and only some differences were observed during ripening, Figure 5A: an increase in the intensity of the ν (O-H) and the relative intensity of the 1705 cm^−1^ shoulder of the ν (C=O) of carboxylic acids involved in weak interactions by H bonding and the presence of new absorptions from phenolic compounds and flavonoids in the 1650-1550 cm^−1^ region and at 834 cm^−1^. The area of this last band, γ(C-H), assigned to the C-H out-of-plane bending vibration of 1,4-disubstituted benzene molecules, was monitored during fruit development, Figure 5B. A significant increase was observed during ripening, starting at mature green and reaching a maximum at red ripe, which was associated with an important accumulation of phenolic compounds. Change of the area of γ(C-H) was barely detected in the cuticle inner surface compared to the cutin and cuticle outer surface, indicating a heterogeneous distribution of phenolic compounds within the cuticle. Figure 5C shows the variation of the cutin esterification index, calculated as the ratio between the intensities of the C=O stretching vibration of the ester (1730 cm^−1^) and the asymmetric stretching vibration of the methylene (2925 cm^−1^) functional groups, during tomato fruit development. Esterification values were high during growth but decreased during ripening, indicating a chemical cleavage of cutin ester bonds. Deconvolution of the C=O stretching region showed that ester groups were transformed into carboxylic acid groups with weak H bonds. The esterification index was related to the area of the γ(C-H) vibration and a linear relationship was observed, Figure 5D. Both parameters showed a significant and negative correlation: an increase in the area of the γ(C-H) was accompanied by a decrease in the esterification index. This correlation suggested that this band can be used to monitor the changes in cutin matrix produced by phenolic compounds. From a different perspective, chemical changes associated with ripening in the tomato fruit surface were studied *in vivo* using a portable spectrometer and a confocal Raman microscope (Trebolazabala et al., 2013). The main compounds identified in mature green tomatoes were cutin and waxes, which were significantly reduced in ripe fruits with the appearance of carotenes, polyphenols and polysaccharides.

In a similar way, the development of different leaves was researched by Ribeiro da Luz (2006). In this study a comprehensive analysis of the spectral differences between the surfaces of young and mature leaves was carried out by ATR-FTIR. Differences in bands ascribed to polysaccharides, amorphous silica, aromatic compounds and cutin were observed during leaf expansion. The 1008 cm^−1^ band, specific of polygalacturonic acid, showed little modification in some species (*Aesculus hippocastanum* L. and *Aesculus octandra* Marsch.) but was increased in others (*Carya ovata* (Mill.) K.Koch, *Cornus florida* L., *Liriodendron tulipifera* L. and others). The band at 1032 cm^−1^ generally ascribed to polysaccharides, was stronger in mature leaves of *Acer rubrum* L., *Quercus alba* L., *Quercus rubra* L. and others than in the corresponding young leaves. These spectral modifications could be related to several changes in the polysaccharide fraction: new compounds, changes in crystallinity, differences in hydrogen bonding, anomeric or positional linkages and/or modifications in the microfibril orientation. *Fagus grandifolia* Ehrh. and *Magnolia grandiflora* L. displayed a broadening and displacement of the 1050 cm^−1^ band during leaf expansion. This absorption was assigned to amorphous silica and indicated an increase of this compound as leaves matured. Interestingly, the band near 840 cm^−1^, attributed to aromatic compounds, showed a different behavior depending on the species, decreased in mature leaves of *Prunus serotina* Ehrh. but increased in those of *Ginkgo biloba* L. Finally, bands around 1727 and 1165 cm^−1^, assigned to ν (C=O) and ν_*a*(C-O-C)_, respectively, are related to the cutin biopolyester. They showed different patterns of variation during leaf expansion depending on the species: stronger in young leaves (*A. rubrum*, *C. ovata*, *C. florida*, etc), weaker in young leaves (*L. tulipifera*, *M. grandiflora*, and *Q*. *alba*) or unchanged (*A. hippocastanum*, *F. grandifolia*, *G. biloba*, etc). Similarly, transition of the ν (C=O) band from a single peak to a doublet with a shoulder at 1716 cm^−1^ (associated with ester groups interacting by H bonds) was also observed in some species during leaf maturation (*A. rubrum*, *A. hippocastanum*, *F. grandifolia* and others). The opposite was true for other species (*A*. *octandra* and *L. tulipifera*). These modifications were justified according to the development of the cutin, with the consequent variations in the esterification index, or other changes in the molecular environments. On the other hand, FT-Raman and ATR-FTIR spectroscopies have also been used to analyze the aging of the surface of spruce needles (Křížová et al., 1999; Plešerová et al., 2001). Mature needles showed a slight decrease of 2934 and 1440 cm^−1^ vibrations associated with saturated aliphatic chains, which was interpreted as a loss of cuticular waxes.

In a broader sense, IR spectroscopy has allowed the characterization of different components during the growth of plant organs. Specifically, the development of the epidermal cell wall of flax hypocotyls was studied by FT-IR microspectroscopy (Stewart et al., 1995). Five days after seed germination, the main peaks of the spectrum were ascribed to proteins (ν (C=O) at 1660 cm^−1^ and δ (N-H) at 1550 cm^−1^ from the amide groups) with some participation of suberin/cutin esters (1740 and 1260 cm^−1^), pectin (1680-1600 and 955 cm^−1^), and lignin (1595 and 1510 cm^−1^). Later on, at 11 days, signals of proteins were reduced in comparison with those of pectin and lignin, while the bands of esters did not change. Finally, at 20 days, some strong new bands at 1510 and 1460-1430 cm^−1^ were assigned to the δ (C-H) of methylene groups, indicating an important deposition of suberin/cutin. Furthermore, the appearance of a strong band at 816 cm^−1^, associated with 1,4-substituted aromatic rings, suggested the combined deposition of aromatic and aliphatic material during this period.

### Interaction with exogenous molecules

The cuticle is the first barrier to overcome by any chemical from the environment before entering the aerial parts of plant. In the same way, the plant cuticle avoids massive loss of water from the plant to the environment. The interaction of these molecules, mostly exogenous chemicals and water, with the plant cuticle has been characterized by IR and, to a lesser extent, Raman spectroscopies.

#### Water-plant cuticle interactions

**Figure 6:**
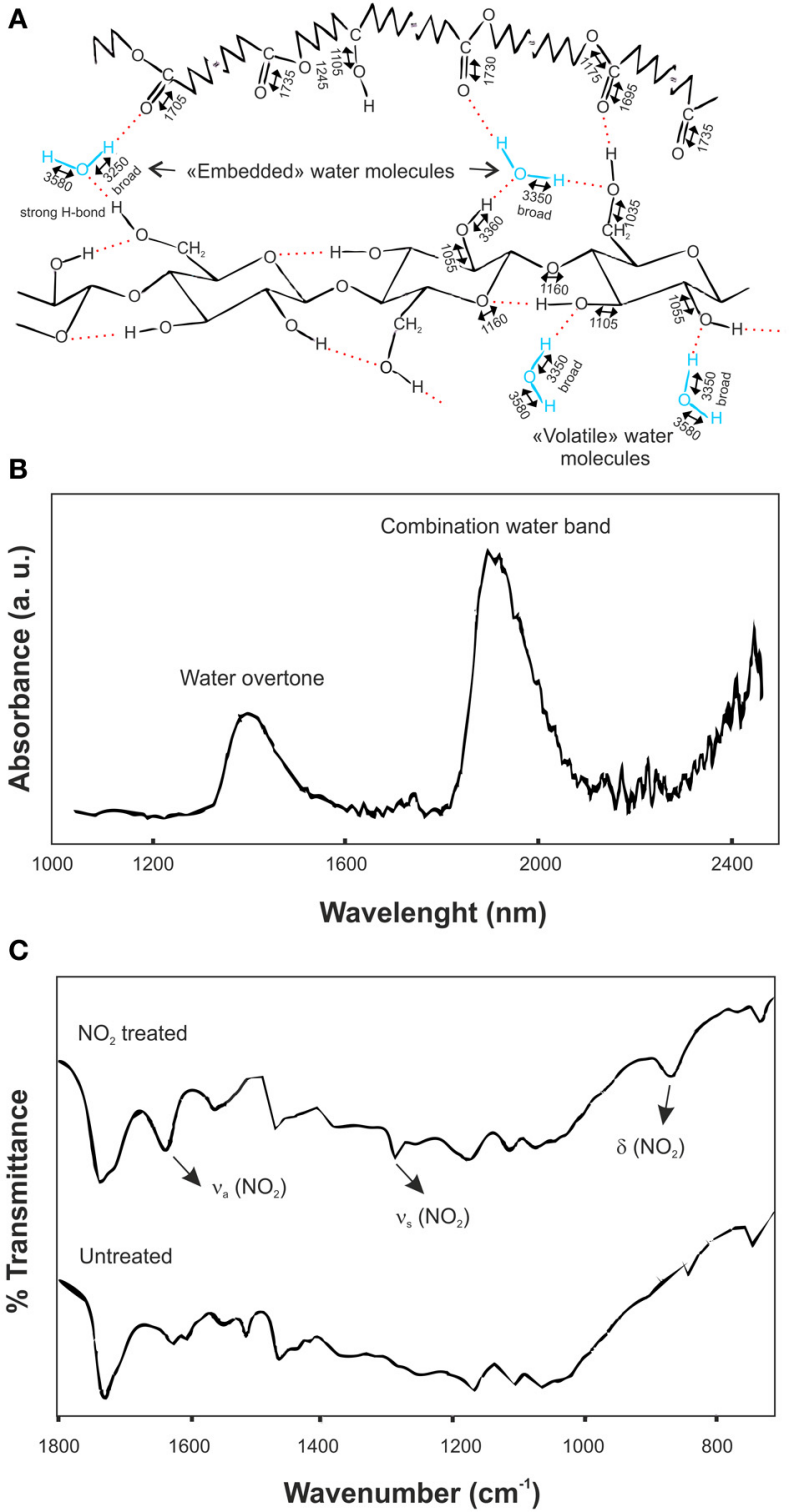
**(A)** Assignment of vibrational bands in an isolated ivy leaf cuticle. Stretching bands are indicated by two-headed arrows above wavenumbers and are rounded to 5 cm^−1^. H_2_O molecules are represented in the “volatile” and “embedded” configurations (adapted from Maréchal and Chamel, 1996, with permission of John Wiley and Sons). **(B)** Difference NIR reflectance spectrum between *Agave americana* decutinized plant cuticles at 98 and 30% relative humidity (adapted from Domínguez and Heredia, 1999, with permission of Elsevier). **(C)** Infrared spectra of NO_2_-treated and untreated isolated tomato fruit cuticular membranes in the 1800-600 cm^−1^ spectral region (adapted from Luque et al., 1994).

IR spectroscopy is a powerful technique to determine the configuration of water molecules in the plant cuticle. These configurations have been identified by evaporation, heating and addition of deuterated water (Maréchal, 1996; Maréchal and Chamel, 1996, 1997). At low water concentration two configurations for H_2_O molecules were defined: “volatile” and “embedded” water molecules. “Volatile” water molecules were in equilibrium with the room moisture and were held by one hydrogen bond formed with the hydroxyl groups of, mainly, polysaccharides. On the other hand, “embedded” molecules participated in the hydrogen bond network of the cuticle, did not evaporate even at temperatures above 100°C, and were held by two strong hydrogen bonds with the cutin and the polysaccharides at the same time or by three of such interactions similar to those described for “volatile” water molecules. Figure 6A summarizes this description. The hydration process of plant cuticles was simulated by addition of deuterated water (D_2_O). Linear combinations of IR spectra before, during and after the hydration revealed two chemical processes: the fixation of D_2_O or HDO molecules inside the plant cuticle and the exchange of O-H groups into O-D groups. In this context, most of deuterated water molecules were in a “volatile” state, but some of them could modify the position of ν (C-O) and ν (C=O) bands, suggesting their penetration inside the cutin matrix. Concerning the H/D exchange, the difference spectra of the sample taken before deposition of the deuterated water droplet and after the submission to a dry atmosphere revealed that O-H were substituted by O-D groups: ν*^H^_s_*(O-H···O) in the region of 3000-3600 cm^−1^ and ν*^H^*(C-O)at 1105 cm^−1^ were reduced, while ν*^D^_s_*(O-D···O) in the region of 2200-2600 cm^−1^ and ν (C-O) at 1085 cm^−1^ were increased.

Similar results were obtained by NIR reflectance, Figure 6B (Domínguez and Heredia, 1999). In this case, the difference spectrum between the decutinized plant cuticles of *A. americana* leaf at 98 and 30% relative humidity was analyzed. The combination band of water molecules in the 1900 nm region showed two relative peaks at 1905 and 1945 nm. The first one was ascribed to water molecules with a non-bonded or a very weakly bonded OH group, while the second one was associated with highly bonded hydroxyl groups, in other words, “volatile” and “embedded” water molecules defined above. Also, an overtone band of water molecules was recorded at 1405 nm, confirming the existence of two different types of OH groups.

#### Chemical-plant cuticle interactions

The cuticle plays an important role in the control of the penetration of herbicides, plant growth regulators and hazardous chemicals, acting as the first barrier to the sorption and uptake of xenobiotics deposited from the atmosphere.

NO_2_ is an air pollutant that shows an irreversible sorption on the plant cuticle by binding with the phenolic components. Figure 6C displays the transmission FT-IR spectra of NO_2_ treated and untreated isolated tomato fruit plant cuticles (Luque et al., 1993, 1994). In the infrared spectrum of the treated cuticle new bands were observed at 1631 and 1278 cm^−1^, ascribed to the asymmetrical and symmetrical stretching vibrations of the NO_2_ group, respectively, and at 860 cm^−1^, associated with the NO_2_ bending vibration. Furthermore, some spectral modifications were identified in the region of the aromatic domain of the plant cuticle. These included a noticeable spectral change around 1620 cm^−1^ (interpreted in terms of different chemical arrangements of specific phenolic compounds after nitrogen oxide treatment), an increase of the band at 1716 cm^−1^ (caused by a shift of the band associated with the keto groups of the phenolic compounds) and small changes around 1550 cm^−1^. Similarly, the interaction of DMSO with isolated cuticles was analyzed by FT-IR (Luque et al., 1994). In this case, the weak bands at 1018 (ν (S=O)), 950 (δ (SCH)), and 714 cm^−1^ (ν (C-S)) were assigned to the different vibrations of the DMSO molecule. The comparison with the liquid DMSO showed that the ν (S=O) was shifted to lower wavenumbers while the wavenumber of the ν (C-S) was increased. Also, the shoulder at 1713 cm^−1^, ascribed to ester groups H-bonding with the hydroxyl groups of the plant cuticle, disappeared. After DMSO desorption, the infrared spectrum of the sample was identical to the spectrum of the untreated cuticle, including the reestablishment of the shoulder at 1713 cm^−1^. The analysis of these data indicated that a specific and reversible interaction occurs between DMSO and some chemical functional groups in the cuticle: DMSO forms an H-bond between the oxygen of the S=O functional group and the hydroxyl groups of the cuticular membrane.

Epicuticular waxes have an important role in the incorporation of pesticides to plants. They constitute the first point of contact with the chemical. The interaction chemical-epicuticular waxes can produce changes in the chemical stability of the exogenous molecule and, hence, in its effectiveness. FTIR spectroscopy can characterize such interactions. For instance, FTIR bands assigned to the nitro group of pesticides fenitrothion and parathion showed significant shifts when were mixed with tomato epicuticular waxes, revealing a strong interaction that modified the photodegradation behavior of the chemicals (Fukushima and Katagi, 2006).

Also, other exogenous material as inorganic particle matter deposited on plant cuticles can be analyzed. In this sense, Raman microspectroscopy was used to analyze the composition of these particles during the foliar lead uptake by lettuce exposed to atmospheric fallouts (Uzu et al., 2010). Raman microspectroscopy allowed the identification of inorganic material rich in mixed carbonates of Ca and Mn, MnO_2_ and PbSO_4_.

### Degradation of plant cuticles

Plant cuticles have a moderately high biological and chemical stability. However, they can be degraded by the action of soil organisms such as fungi and bacteria. Decomposition of plant cuticles isolated from tomato fruits, pepper fruits and citrus leaves incubated in soil was characterized by DRIFT (Chefetz, 2007). Main modifications in the spectra were a decrease of the absorptions at 2930 and 2850 cm^−1^ (asymmetric and symmetric stretching of the methylene groups, respectively) and a reduction of the 1740-1730 cm^−1^ vibration (C=O stretching of ester groups). The ratio of the intensities 2930:1730 remained constant for tomato samples and was slightly increased with time for the cuticles of pepper and citrus. These data suggested that cutin was continuously decomposed in soil, while the fraction of cutan was practically unaltered.

On the other hand, IR can also be employed to analyze minor degradation. In this sense, the effects caused by low dose γ-irradiation in tomato fruit cuticles were identified by ATR-FTIR (Heredia-Guerrero et al., 2012). Samples after the irradiation showed an intense reduction of the asymmetric and symmetric stretching vibrations of the methylene groups, 2917 and 2848 cm^−1^ respectively, while the C=O stretching region (1800-1650 cm^−1^), associated with the cutin, remained unaltered, indicating a partial removal of epicuticular waxes of these samples.

### Fossilized plant cuticles

Plant cuticles are frequently preserved in organic fossils (Almendros et al., 1999). Fossil cuticles are minor constituents in coals and coaly shales, but major components in some organic deposits (Kerp, 1991). Many studies on the characterization of fossilized plant cuticles by IR spectroscopy have been carried out (e.g., Lyons et al., 1995; Zodrow et al., 2000, 2009; 2012a,b; D'Angelo, 2006; Zodrow and Mastalerz, 2009; D'Angelo et al., 2010, 2011; D'Angelo and Zodrow, 2011 and others). In these studies an improvement of the assignments was achieved by calculation of area ratios. This allowed a better characterization of the chemical nature and composition of fossilized cuticles. In the Table 2 a set of such area ratios are shown (Zodrow et al., 2012a).

**Table 2:** **Definition of semi-quantitative ratios from FTIR and their interpretation for the characterization of fossilized plant cuticles (Zodrow et al., 2012a)**.

**Ratio**	**Band region (cm^−1^) Band-region ratios**	**Interpretation and remarks**
CH_2_/CH_3_	3000-2800	Methylene/methyl ratio: It relates to aliphatic chain length and degree of branching of aliphatic side groups. Higher value implies comparatively longer and straight chains, a lower value, shorter and more branched chains. In some cases, CH_2_ and CH_3_ groups attached to aromatic rings can contribute and produce wrong results
Al/Ox	(3000-2800)/(1800-1600)	Aliphatic/oxygen-containing compounds ratio: Relative contribution of aliphatic C-H stretching bands (Al) to the combined contribution of oxygen-containing groups and aromatic carbon (Ox). From higher values decreasing oxygen-containing groups can be inferred. This ratio could provide some information about oxidation in organic matter
C=O/C=C	(1700-1600)/(1600-1500)	Carbonyl/aromatic carbon groups ratio: Relative contribution of C=O to aromatic carbon groups. Higher values indicate increasing carbonyl/carboxyl groups to aromatic carbon groups
C=O cont	(~1714)/(1800-1600)	Carbonyl contribution: Relative contribution of carbonyl/carboxyl groups (C=O; peak centered near 1714 cm^−1^) to combined contribution of oxygen-containing groups and aromatic carbon (C=C) structures
Ar/Al	(900-700)/(3000-2800)	Aromatic C-H out-of-plane bending/aliphatic ratio: Contribution of aromatic C-H out-of-plane bending modes to aliphatic C-H stretching bands (aliphatic H bands). Higher values indicate higher aromaticity in the organic matter
Ar/ C=C	(900-700)/(1600-1500)	Aromatic C-H out-of-plane bending/aromatic carbon groups ratio: Ratio of integrated area of aromatic C-H out-of-plane bending deformations to those of aromatic carbon groups. Used as measure of degree of condensation of aromatic rings

Additionally, FTIR spectroscopy has been used to compare cuticles from fossil and extant plants, showing a transformation during the fossilization of ester groups to carboxylic acid or ketone functional groups (Mösle et al., 1998). Recently, a fossil cutin characterized by strong peaks of ester C=O groups (1730-1715 cm^−1^) and aromatic C=C absorptions at 1640-1645 cm^−1^ has been recorded (D'Angelo et al., 2013). The comparison of the esterification indexes of this cutin and tomato fruit cutin indicated a similar cross-linking degree of the polymeric structure for the fossil and extant taxa.

### Chemical imaging

IR and Raman chemical imaging techniques can provide important information about the distribution of different compounds in plant samples with a high chemical selectivity and a good spatial resolution.

NIR-Raman microspectroscopy and CARS microscopy were used to map triterpenoid and aliphatic distribution in isolated cuticles from the adaxial and abaxial sides of cherry laurel (*Prunus laurocerasus* L.) leaves (Yu et al., 2008). The Raman peak at 728 cm^−1^ corresponding to the C-C stretching in the ring vibration of triterpenoids was used to monitor the distribution of these cyclic components. In addition, the peak at 1130 cm^−1^ can be used to map very long chain constituents. Raman maps of the adaxial cuticle showed that aliphatic waxes were homogeneously distributed, while the triterpenoids were preferentially located on the periclinal regions of the pavement cells. In the abaxial cuticles, triterpenoids were found in higher amounts on the guard cells. Aliphatic compounds accumulated in the cuticle above the anticlinal cell walls of the pavement cells. In a similar way, CARS microscopy was used to analyze the epicuticular waxes of *P. laurocerasus*, *Hoya carnosa* (L.f.) R.Br. and *Monstera deliciosa* Liebm leaves (Weissflog et al., 2010). In this case, most strong bands in the Raman spectrum of extracted epicuticular waxes, asymmetrical and symmetrical stretching vibrations at around 2880 and 2840 cm^−1^, respectively, were employed to record CARS images. Results demonstrated the feasibility to monitor epicuticular waxes by means of CARS microscopy and were comparable with s.e.m. micrographs.

**Figure 7:**
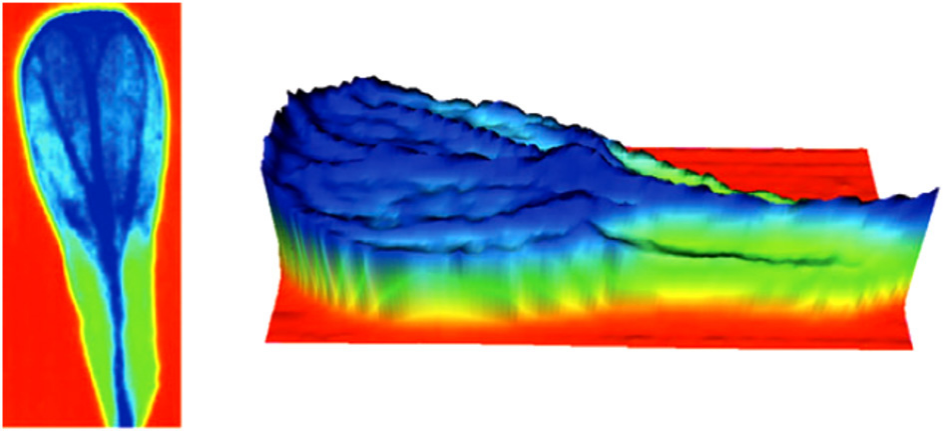
**FTIR imaging of Arabidopsis petals: absorbance maps obtained on the basis of the second derivative of spectra for esters of fatty acids at 1734 cm^−1^**. More negative intensities (high concentration) are indicated in dark blue. The zero level is indicated in red (adapted from (Mazurek et al., 2013), with permission of John Wiley and Sons).

FTIR imaging technique have been used to obtain absorption maps of cell wall aliphatic polyesters (cutin), amides and polysaccharides of Arabidopsis petals (wild type and cutin mutants) by transmission Fourier transform infrared microspectroscopy, Figure 7 (Mazurek et al., 2013). In this study, the absorbance maps obtained on the basis of the second derivative of bands associated with C-H (2928, 2919, 2850, and 1465 cm^−1^) vibrations showed a distribution of aliphatic material dominant in the petal lamina, with intensities twofold smaller in the hinge region. These maps were very similar to the corresponding for ester band (1734 cm^−1^), indicating that cutin is the origin of these vibrations.

## Concluding remarks and outlooks

The characterization of plant cuticles by IR and Raman spectroscopies has provided significant and valuable information about the chemical nature, structure and arrangement of the different cuticle components. Nonetheless, it is important to remark that despite the advantages of IR and Raman spectroscopies, the information acquired from them is often limited. In our opinion, it is highly advisable the complementation of IR and Raman results with other techniques. In this sense, the chemical characterization by nuclear magnetic resonance and the compositional analysis by gas chromatography of the plant cuticle components are appropriate tools.

It is important to remark that many of the reviewed papers are focused in the characterization of tomato fruit cuticles. This notoriety can be justified by the elevated agronomic interest of these fruits, their high proportion of cuticle material, and the easy isolation of their cuticles. Besides, the tomato fruit cuticle is considered as a model system with well-known composition, structure and properties. The direct extrapolation of results to the cuticles of other species and/or organs could be wrong. This could be a good starting point in the research of other cuticle systems by IR and Raman spectroscopies.

Several promising outlooks are envisaged in the research on plant cuticles. Confocal Raman microscopy approaches used in the characterization of cell walls can be extrapolated to plant cuticles (Gierlinger et al., 2010, 2012). This can be applied, for instance, to the study of the plant cuticle development and decomposition. Also, the screening of plant cuticle mutants could be carried out by Raman and IR analysis similarly to other studies in cell walls (Chen et al., 2008). On the other hand, the exogenous chemical-plant cuticle interaction remains as an interesting topic and the application of models to study the diffusion of molecules through the cuticle by ATR-FTIR can be useful and interesting (Fieldson and Barbari, 1992; Yi and Pellegrino, 2002).

Finally, in terms of Ludwig Wittgenstein (see Initial quotation), IR and Raman spectroscopies have shed light on the *internal properties* of plant cuticles. In other words, they have allowed a better comprehension of the cuticular membrane. However, more lights are necessary in the understanding of this heterodox and complex plant system.

### Conflict of interest statement

The authors declare that the research was conducted in the absence of any commercial or financial relationships that could be construed as a potential conflict of interest.

## Abbreviations

FTIR: Fourier Transform Infrared
ATR: Attenuated Total Reflection
DRIFT: Diffuse Reflectance Infrared Fourier Transform
NIR: Near Infrared
CARS: Coherent Anti-Stokes Raman Spectroscopy
TIR: Total Internal Reflection
DMSO: dimethyl sulfoxide.
